# Supplementary material for: Human Vγ9Vδ2 T cells exhibit antifungal activity against Aspergillus fumigatus and other filamentous fungi
Source: Microbiol Spectr. 2024 Mar 1;12(4):e03614-23. doi: 10.1128/spectrum.03614-23 (PMC10986472; doi:10.1128/spectrum.03614-23)
Supplement: Supplemental Fig. S1 to S4 — Profiles of Vγ9Vδ2 T cells. [file spectrum.03614-23-s0001.pdf]

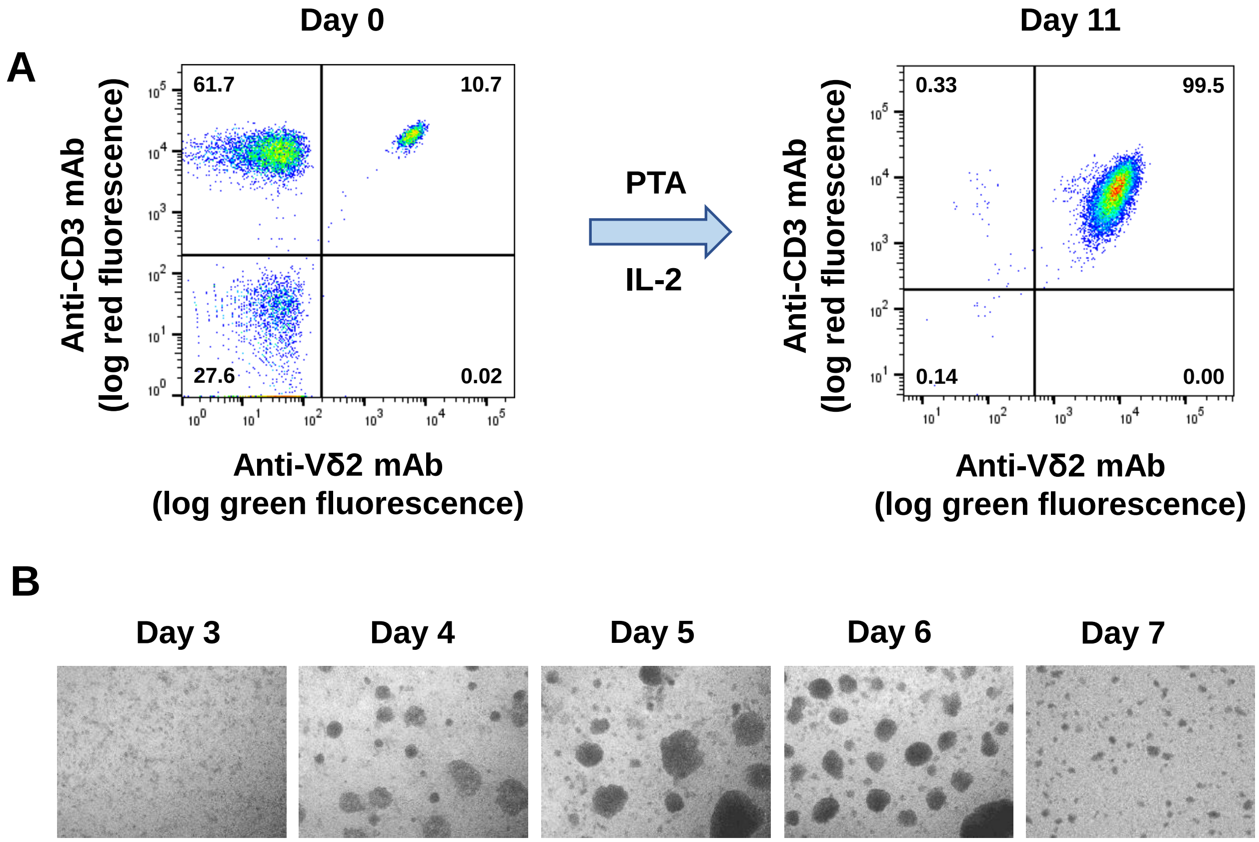

Supplementary Fig. 1. Expansion of Vδ2$^+$ T cells by PTA and IL-2. (A) Flow cytometric analysis of PTA/IL-2-mediated expansion of Vδ2$^+$ T cells derived from a healthy donor. Peripheral blood (10 mL) was drawn from a healthy adult volunteer. The blood sample was diluted with 10 mL of PBS, which was loaded onto 20 mL of Ficoll-Paque^{TM} PLUS (Cytiva, Shinjuku-ku, Tokyo, Japan) in a 50 ml conical tube (Corning Inc., Corning, NY), which was centrifuged at 1,700 rpm (590 x g) and ambient temperature for 30 min. The fluffy layer was then collected and the peripheral blood mononuclear cells (PBMC) were washed twice with 35 mL and 13 mL of PBS. The cell pellets were resuspended in 7.2 ml of Yssel's medium supplemented with 10% human AB serum (Yssel, H., *et al.* Serum-free medium for generation and propagation of functional human cytotoxic and helper T cell clones. ***J. Immunol. Methods*** 72:219-227, 1984. doi: 10.1016/0022-1759(84)90450-2). To the PBMC suspension (6 mL) was added 6 μL of 1 mM PTA in DMSO, which was dispensed into 4 wells of a 24-well plastic plate (Corning Inc.). After the cell suspension was incubated at 37 ^{o}C with 5% CO_2 overnight, 100 IU/mL of IL-2 was added to each well. On day 2, the culture supernatants were replaced with 1.5 mL each of Yssel's medium, to which was added 100 IU/mL each of IL-2. On days 3 and 4, additional 100 IU/mL of IL-2 was added to each well. On day 5, 1.5 mL each of Yssel's medium was added to each well and the respective cell suspension was split into 2 wells each (a total of 8 wells), to which was added 100 IU/mL of IL-2. On day 6, the cell suspensions were transferred into a 75 cm^2 plastic culture flask (AGC Techno Glass Co., Ltd., Haibara-gun, Shizuoka, Japan) and Yssel's medium was added to give a total volume of 30 mL. On day 7, the cell suspension was split into three 75 cm^2 plastic culture flasks. On days 8 and 9, the culture volume was increased by 2-fold up to a total of 12 flasks. On day 10, the cells were harvested and the cell number was counted. Before and after expansion with PTA/IL-2, the cells were stained with phycoerythrin (PE)-labeled anti-CD3 mAb and fluorescein isothiocyanate (FITC)-labeled anti-Vδ2 mAb and analyzed through a FACS Lyric flow cytometer (BD Biosciences, Franklin Lakes, NJ). **(B) PTA-mediated clustering of Vδ2$^+$ cells.** After stimulation with PTA/IL-2, the cell clustering was monitored under a microscope equipped with a CCD camera (Olympus Corp., Shinjuku-ku, Tokyo, Japan).

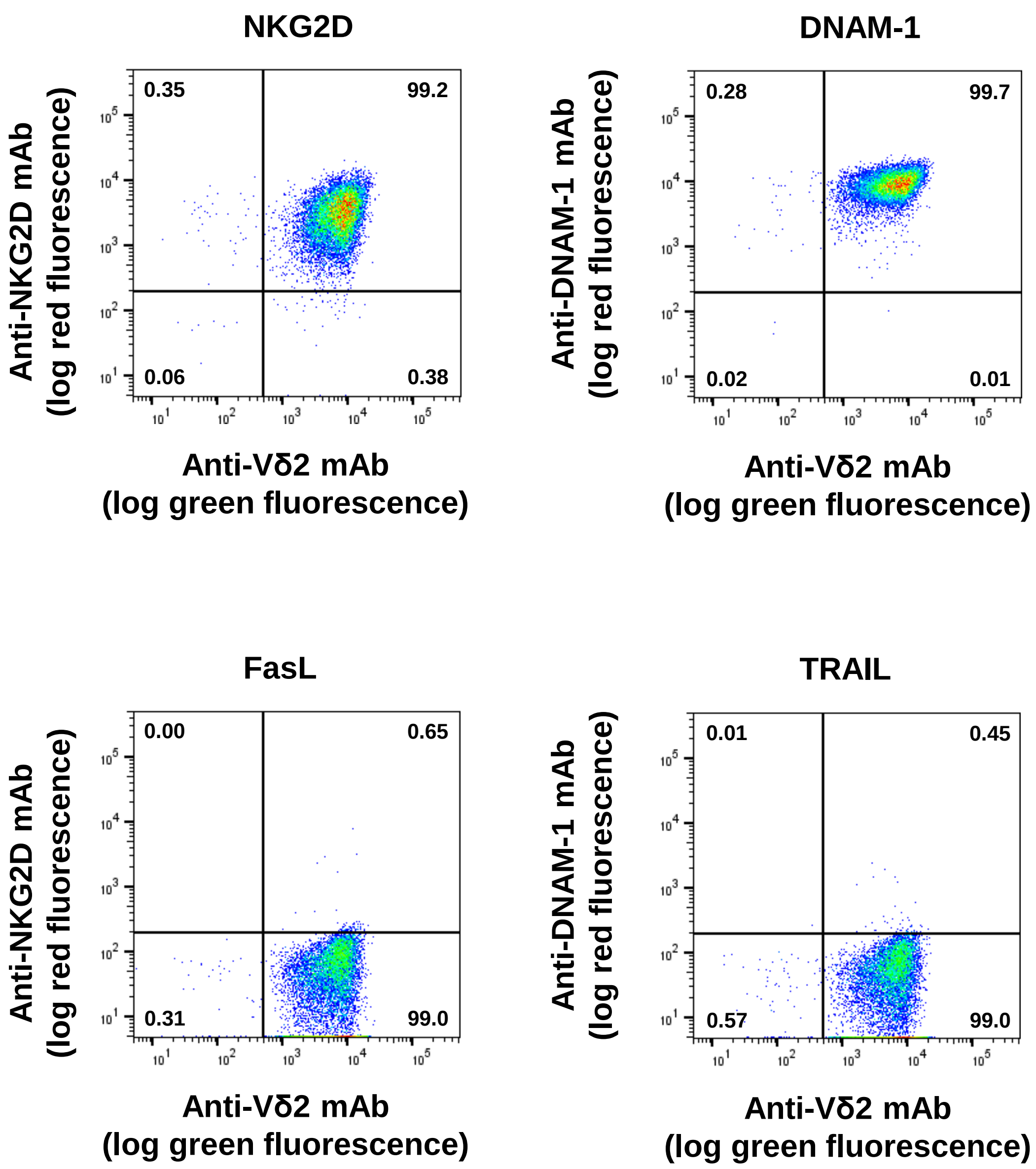

Supplementary Fig. 2. Flow cytometric analysis of natural killer cell markers on PTA/IL-2-mediated expansion of Vγ9Vδ2 T cells derived from a healthy donor. After expansion by PTA/IL-2 for 11 days, Vγ9Vδ2 T cells were stained with phycoerythrin (PE)-labeled anti-NKG2D or DNAM-1 mAb and fluorescein isothiocyanate (FITC)-labeled anti-Vδ2 mAb and analyzed through a FACS Lyric flow cytometer (Becton Dickinson and Co., Franklin Lakes, NJ). The cell population was visualized using a FlowJo software ver. 10 (FlowJo LLC, Ashland, OR).

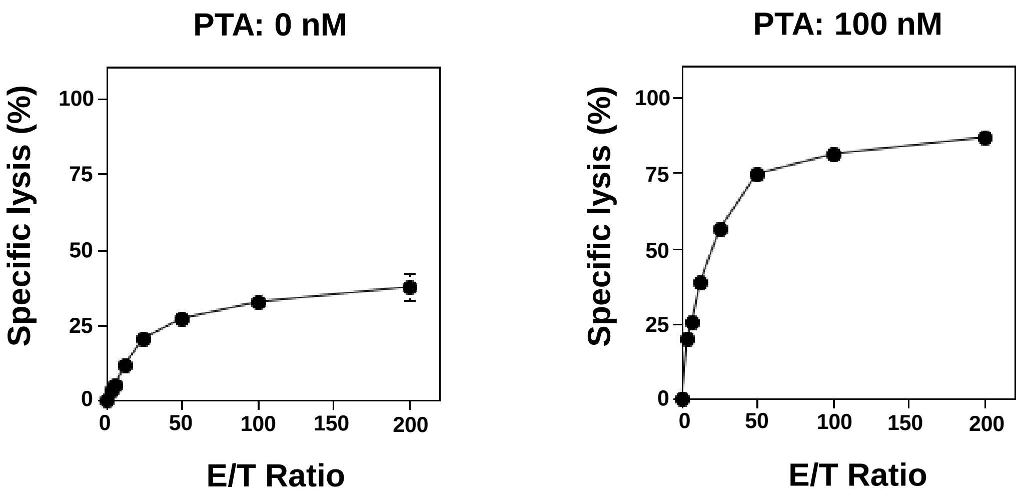

Supplementary Fig. 3. Natural killer cell-like cytotoxicity and TCR-dependent cellular cytotoxicity exhibited by PTA/IL-2-expanded Vγ9Vδ2 T cells derived from a healthy donor. PTA/IL-2-expanded Vγ9Vδ2 T cells were examined for cytotoxic activity against Raji, a human Burkitt's lymphoma cell line. Raji cells (1 x 10^6 cells in 1 mL RPMI1640 medium) were pretreated with 25 μM bis(butyryloxymethyl) 4'-(hydroxymethyl)-2,2':6',2''-terpyridine-6,6''-dicarboxylate (BM-HT, Techno Suzuta Co., Ltd.) at 37 °C for 15 min. After BM-HT was internalized into tumor cells, the pro-chelate reagent was hydrolyzed by intracellular esterases to yield 4'-(hydroxymethyl)-2,2':6',2''-terpyridine-6,6''-dicarboxylate (HT). After the cells were washed three times with 5 mL of RPMI140 medium, Raji cell pellets were resuspended in 20 mL of RPMI1640 medium. Raji cell suspensions (5 x 10^3 cells/100 μL) were dispensed into a 96-well round-bottom plastic plate (Corning Inc.), to which were added 100 μL of a serial dilution of γδ T cells at effector-to-target ratios of 200:1, 100:1, 50:1, 25:1, 12.5, 6.25, 3.125 and 0. The plate was centrifuged at 50 x g for 2 min in a refrigerated centrifuge (Tomy Seiko Co., Ltd., Nerima-ku, Tokyo, Japan) and then incubated at 37 °C with 5% CO_2 for 40 min. Detergent (Techno Suzuta Co., Ltd.) was added to wells at a final concentration of 5 x 10^{-5} M for maximum release and the plate was incubated at 37 °C for 20 more min. After the cell suspensions were mixed using microtips, the plate was centrifuged at 590 x g for 2 min and the supernatants (25 μL each) were transferred into a new 96-well round bottom plate (Corning Inc.) containing 250 μL of europium (Eu^{3+}) solution in 0.3 M sodium acetate buffer, pH 4 (Techno Suzuta Co., Ltd.). The culture supernatant/Eu mixtures (200 μL each) were transferred to a 96-well optical plate (Thermo Fisher Scientific Inc., Waltham, MA). Time-resolved fluorescence (TRF) was measured through a NIVO multi-plate reader (Revvity, Yokohama, Kanagawa, Japan). All measurements were performed in triplicate. Specific lysis (%) was calculated as 100 x [experimental release (counts) – spontaneous release (counts)]/[maximum release (counts) – spontaneous release (counts)]. To examine the effect of PTA on the cellular cytotoxicity, Raji cells were pulsed with 0 nM or 100 nM PTA at 37 °C for 2 h before being pulsed with BM-HT.

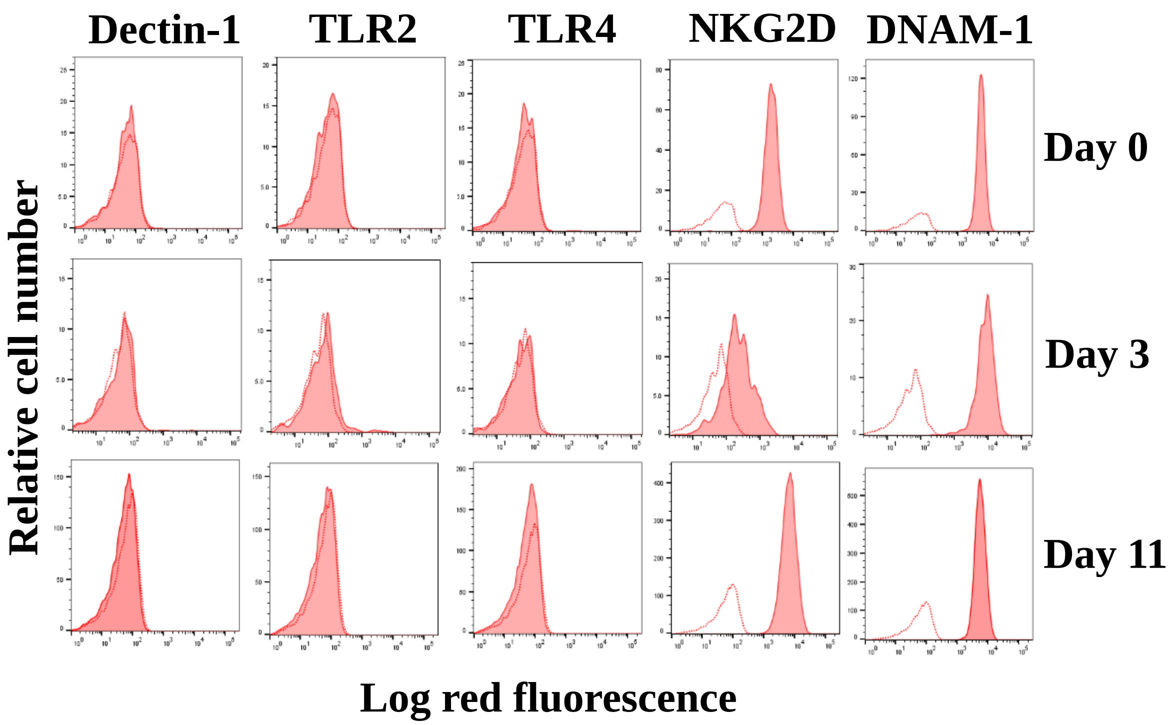

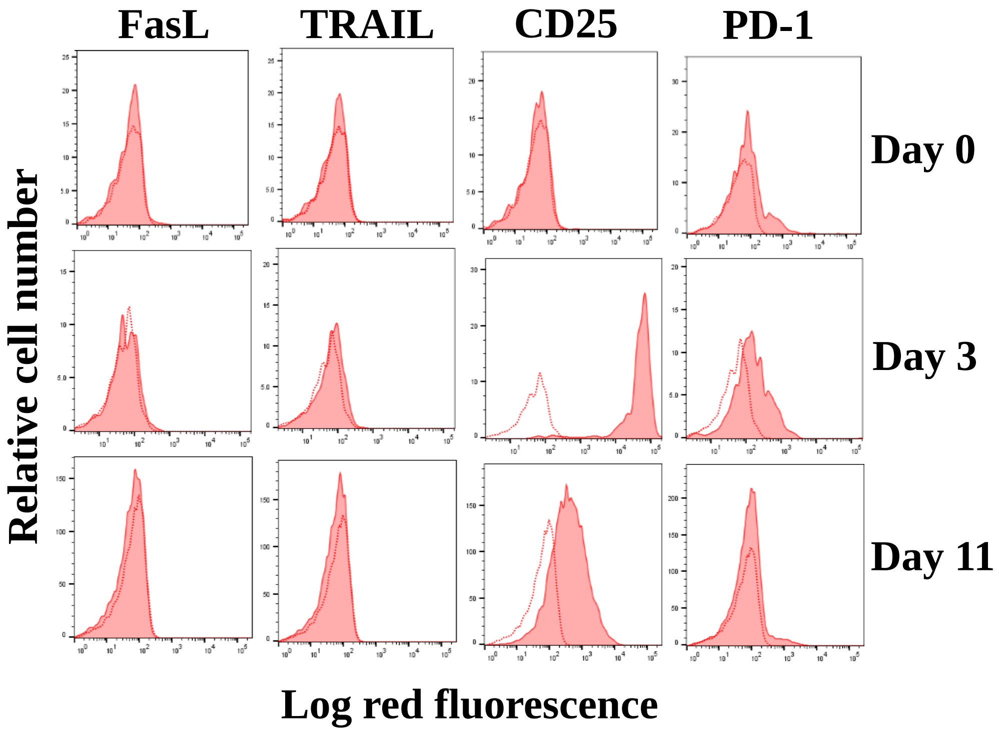

Supplementary Fig. 4. Flow cytometric analysis of several cell markers on Vγ9Vδ2 T cells derived from the same donor on the first day, last day, and the third day of expanded culture. On the first day, last day, and the third day of expanded culture, Vγ9Vδ2 T cells were stained with phycoerythrin (PE)-labeled anti-Dectin-1 (BioLegend), anti-TLR2 (BioLegend), anti-TLR4 (BD Biosciences), anti-NKG2D, anti-DNAM-1, anti-FasL, anti-TRAIL, anti-CD25 and anti-PD-1 mAb and fluorescein isothiocyanate (FITC)-labeled anti-Vδ2 mAb and analyzed through a FACS Lyric flow cytometer (Becton Dickinson and Co., Franklin Lakes, NJ). The cell population was visualized using a FlowJo software ver. 10 (FlowJo LLC, Ashland, OR).
