# Supplementary material for: Human Vγ9Vδ2 T cells exhibit antifungal activity against Aspergillus fumigatus and other filamentous fungi
Source: Microbiol Spectr. 2024 Mar 1;12(4):e03614-23. doi: 10.1128/spectrum.03614-23 (PMC10986472; doi:10.1128/spectrum.03614-23)
Supplement: Supplemental methods — Derivation of Vγ9Vδ2T cells and NK cells. [file spectrum.03614-23-s0002.docx]

**Supplementary Methods**

**Derivation of Vγ9Vδ2T cells**

Peripheral blood samples were treated with sodium heparin (Mochida Pharmaceutical, Co., Ltd., Shinjuku-ku, Tokyo, Japan; 1/100 volume) and diluted with equal volumes of Dulbecco’s phosphate-buffered saline (PBS) (-) (Nissui Pharmaceutical Co., Ltd., Minato-ku, Tokyo, Japan). The diluted blood (20 mL) was loaded onto 20 mL Ficoll-Paque^TM^ PLUS (GE Healthcare BioSciences AB, Chicago, IL, USA) in a 50 mL conical tube (Corning Inc., Corning, NY, USA) and centrifuged at 600 × g and 25 °C for 30 min. The fluffy layer was then transferred into a new 50 mL conical tube and diluted with 2.5 volumes of PBS (-). The PBMC suspension was centrifuged at 900 × g at 4 °C for 10 min. After the supernatant was aspirated, cell pellets were dispersed by tapping and then resuspended in 13 mL PBS (-). The cell suspension was centrifuged at 600 × g at 4 °C for 5 min, and the supernatant was aspirated. Subsequently, the cell pellets were dispersed by tapping and resuspended in 7 mL of Yssel’s medium comprising Iscove’s modified Dulbecco’s medium (Thermo Fisher Scientific, Waltham, MA, USA) supplemented with 10% human AB serum (Cosmo Bio Co., Ltd., Koto-ku, Tokyo, Japan), 3.6 × 10^−2^ M NaHCO_3_ (Nacalai Tesque Inc., Nakagyo-ku, Kyoto, Japan), 3.3 × 10^−5^ M 2-aminoethanol (Nacalai Tesque Inc., Kyoto, Japan), 40 mg/L transferin apo form (Nacalai Tesque Inc., Kyoto, Japan), 5 mg/L human recombinant insulin (Merck & Co., Inc., Darmstadt, Hesse, Germany), 2 mg/L linoleic acid (Merck & Co., Inc., Darmstadt, Germany), 2 mg/L oleic acid (Merck & Co., Inc., Darmstadt, Germany), 2 mg/mL palmitic acid (Merck & Co., Inc., Darmstadt, Germany), 100 μg/mL streptomycin, and 100 U/mL penicillin. The PBMC suspension (1.5 mL each, 1–2.5 × 10^6^ cells/mL) was dispensed into the wells of a 24-well plate (Corning Inc., Corning, NY, USA). A stock solution of PTA in dimethyl sulfoxide (1.5 μL, 1 mM) was added to each well for a final PTA concentration of 1 μM. The plate was incubated at 37 °C and 5% CO_2_ overnight. IL-2 (Shionogi Pharmaceutical Co., Ltd., Chuo-ku, Osaka, Japan) was added to each well daily from Day 1 to Day 4 for a final concentration of 100 U/m. On Day 5, 1.5 mL of fresh Yssel’s medium was added to the wells and mixed by pipetting. Half of the suspension was transferred into new wells, to which 100 U/mL of IL-2 was added. On Day 6, the cell suspension was transferred into a 75 cm^2^ flask (AGC Techno Glass Co., Ltd., Haibara-gun, Shizuoka, Japan). On day 7, the cell suspension was split into three 75 cm^2^ flasks and complete RPMI 1640 medium (Merck & Co., Inc., Darmstadt, Germany) supplemented with 10% fetal calf serum (FCS, Merck & Co., Inc., Darmstadt, Germany), 10^−5^ M of 2-mercaptoethanol (Wako Pure Chemical Industries, Ltd., Osaka, Japan), 100 μg/mL streptomycin (Meiji Seika Pharma Co., Ltd., Tokyo, Japan), 100 U/mL penicillin (Meiji Seika Pharma Co., Ltd.), and 100 IU/mL of IL-2 was added. Vγ9Vδ2 T cells were expanded using complete RPMI1640 medium plus 100 U/mL of IL-2 by Day 11, harvested by centrifuging at 590 × g and 4 °C for 5 min, resuspended in cryopreservation media, dispensed into cryovials, incubated overnight at −80 °C, and then stored in liquid nitrogen until used. Cells collected on days 0 and 11 were stained with 3 μL of phycoerythrin-conjugated anti-CD3 monoclonal antibodies (mAb) (BD Biosciences, San Diego, CA), NKG2D, or DNAM-1 mAb (BioLegend, San Diego, CA) and with fluorescein isothiocyanate (FITC)-conjugated anti-TCR Vδ2 mAb (BD Biosciences) in 50 μL of PBS (-) containing 2% FCS on ice for 15 min in a 96-well round-bottom plate. After three washes with 200 μL of PBS (-)/2% FCS, the cells were resuspended in 200 μL of PBS (-)/2% FCS and analyzed through a FACSLyric flow cytometer (Becton Dickinson & Co., Franklin Lakes, NJ). The cell population was visualized using a FlowJo software ver. 10 (FlowJo LLC, Ashland, OR).

**Derivation of human NK cells**

To PBMC suspensions in PBS containing 0.5% BSA and 2 mM EDTA (MACS buffer) was added a one-fourth volume of anti-CD3 MACSBeads (Miltenyi Biotec, Auburn, CA), which was placed at 4^o^C for 15 min. Two volumes of MACS buffer were added to the PBMC/MACSBeads suspension, which was centrifuged at 300 x g and 4^o^C for 10 min. After the supernatant was aspirated, cell pellets were resuspended in 2 mL of MACS buffer and applied to LD Column (Miltenyi Biotec) equipped with a magnet holder (Miltenyi Biotec), which had been equilibrated with 2 mL of MACS buffer. CD3-negative cells were allowed to be eluted with 2 x 1 mL of MACS buffer into a 15 mL conical tube (AGC Techno Glass Co., Ltd.), to which was added 6 mL of Yssel’s medium containing human AB serum (Cosmo Bio Co., Ltd.). After the tube was centrifuged at 600 x g and 4^o^C for 5 min, the supernatant was aspirated and CD3-negative cells were resuspended in Yssel’s medium to give a cell concentration of ca. 2 x 10^6^ cells/mL, to which were added 100 U/mL of IL-2 (Shionogi Pharmaceutical Co., Ltd., Chuo-ku, Osaka, Japan) with or without 100 ng/mL of IL-18 (Techno Suzuta Co., Ltd.) The cell suspensions were placed into a 24-well plate (Corning Inc., Corning, NY) and incubated at 37^o^C with 5% CO_2_. After 10 days of incubation, NK cells were harvested and analyzed for their anti-fungal activity.
